# Supplementary material for: Identification of Insulin Resistance Biomarkers in Metabolic Syndrome Detected by UHPLC-ESI-QTOF-MS
Source: Metabolites. 2022 Jun 1;12(6):508. doi: 10.3390/metabo12060508 (PMC9227428; doi:10.3390/metabo12060508)
Supplement: Supplementary file 1 [file metabolites-12-00508-s001.zip › metabolites-1736789-supplementary.pdf]

Supplementary supporting Data

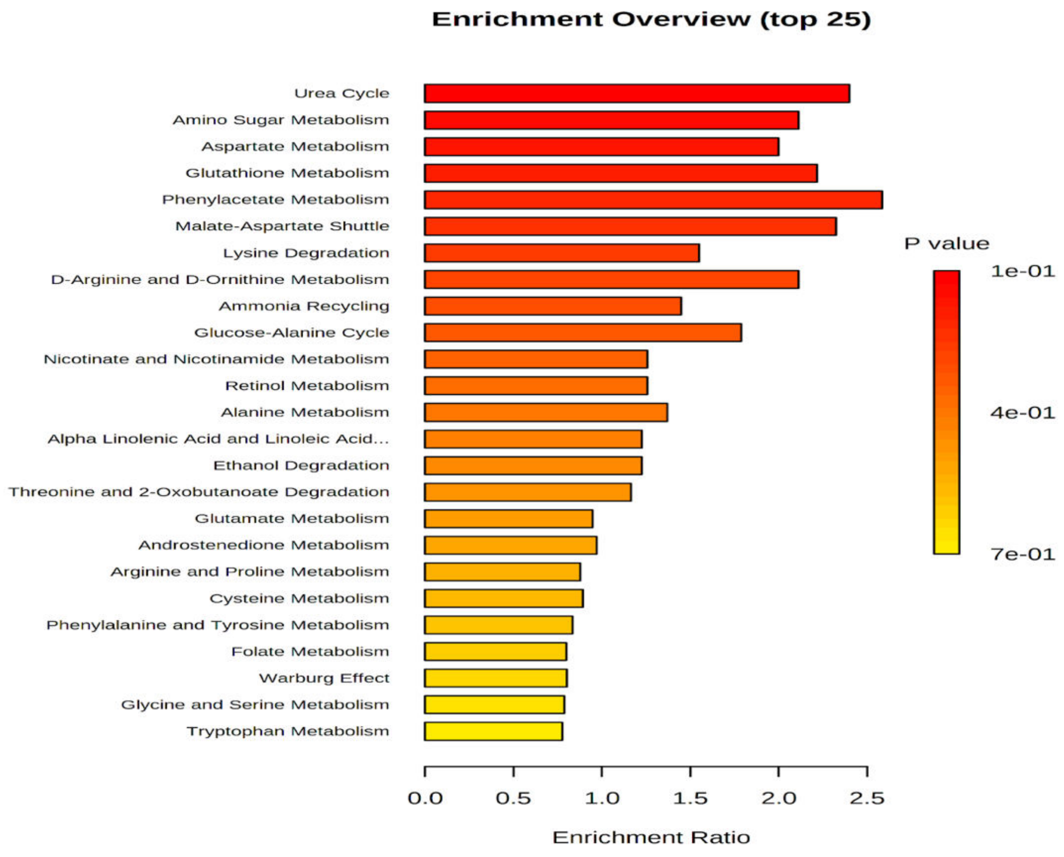

Figure S1: (A) Enrichment Analysis of Controls and Normoglycemic MetS

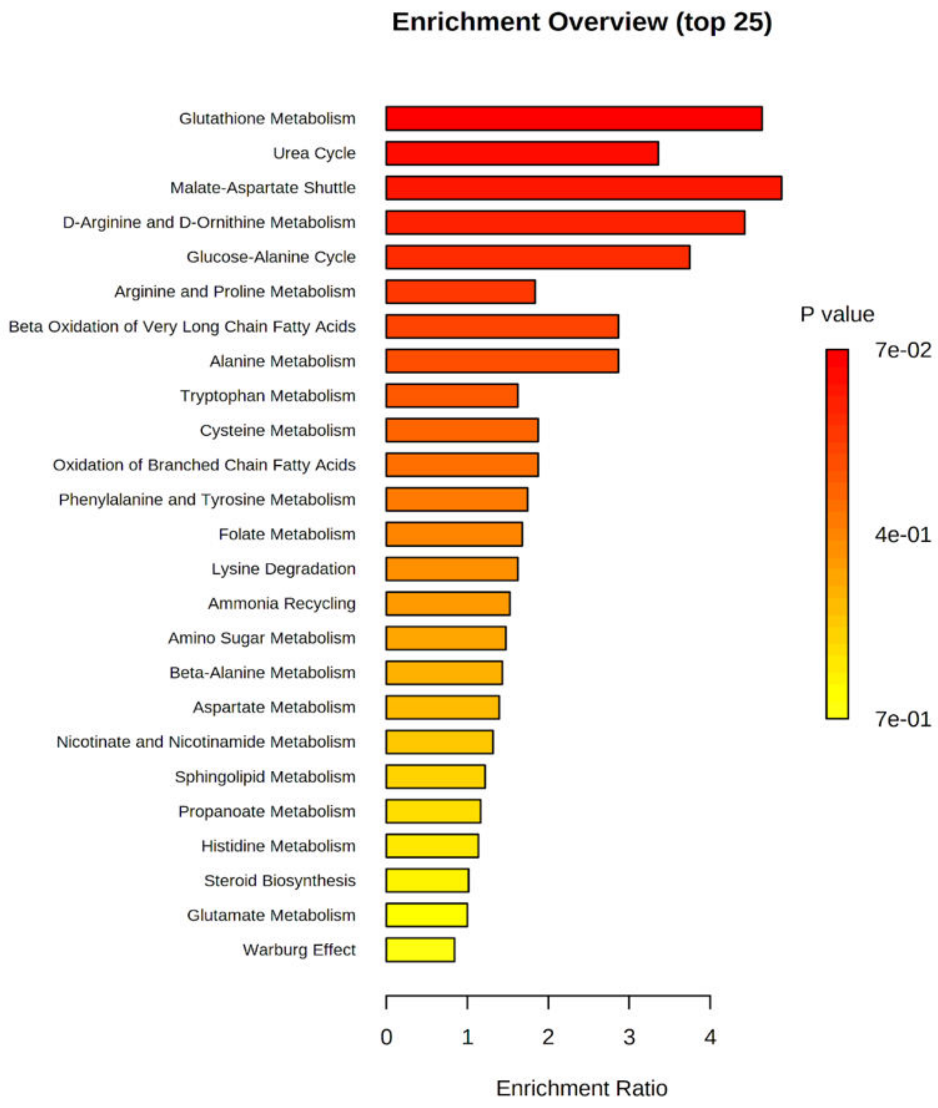

Figure S1: (B) Enrichment Analysis of Normoglycemic MetS and Prediabetic MetS

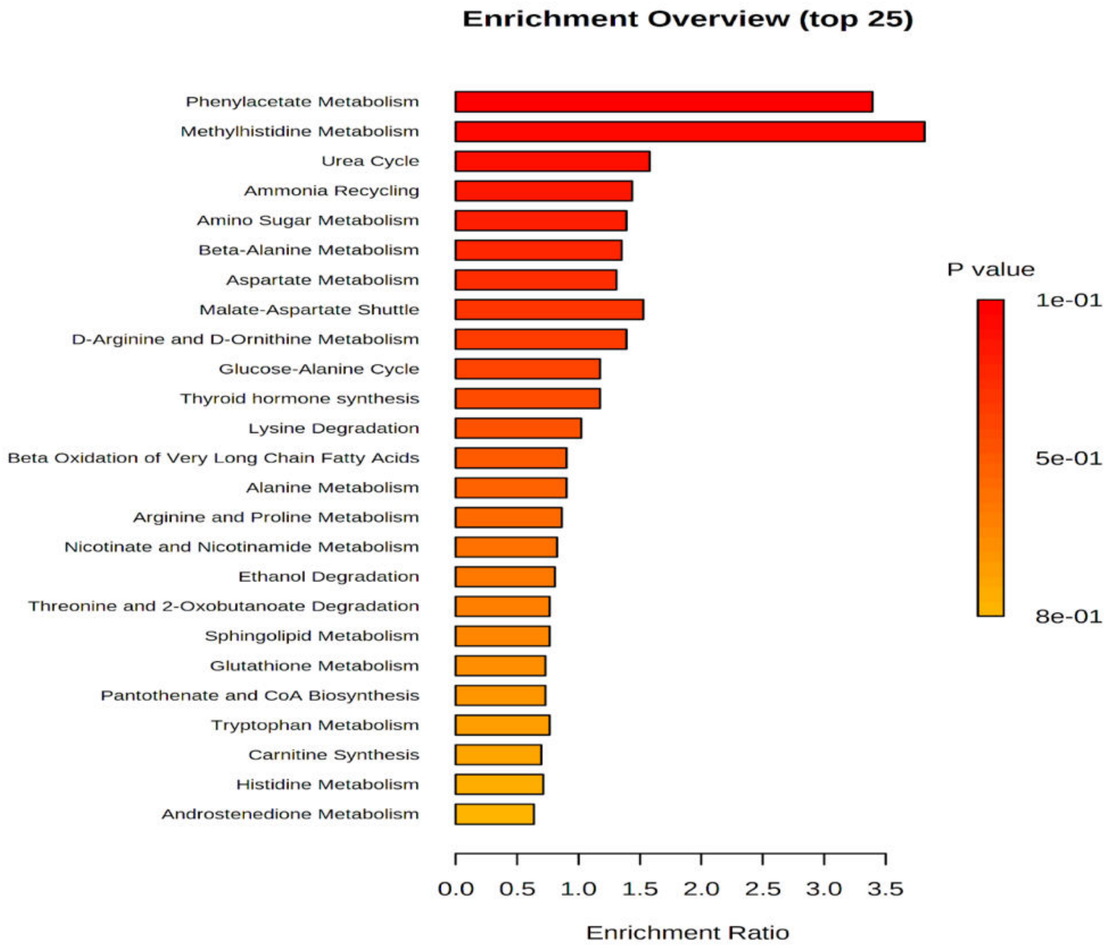

Figure S1: (C) Enrichment Analysis of Controls and Prediabetic MetS

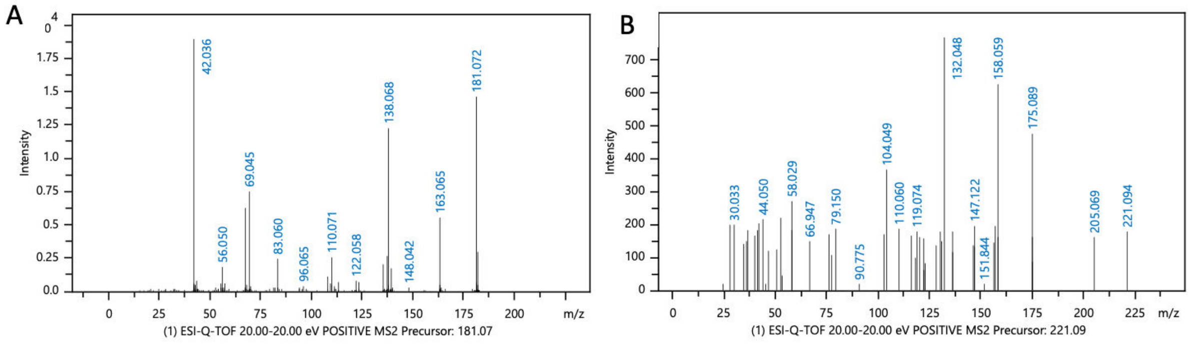

Figure S2: Mass spectra for L-sorbose **(A)** and 5-Hydroxy-L-tryptophan **(B)**

Table S1: Retention time and MS/MS

Name	RT [min]	m/z measured	Mass measured
**1,3-Dimethyluric acid**	**9.75**	**197.06728**	**196.06**
**1-Methylhistidine**	**1.85**	**170.09301**	**169.08574**
**17-Hydroxyprogesterone**	**22.39**	**331.22326**	**330.21598**
**2,4-Diaminobutyric acid**	**17.41**	**119.08565**	**118.07837**
**2-Furoylglycine**	**3.34**	**170.04212**	**169.03485**
**2-Phenylbutyric acid**	**13.42**	**147.08064**	**164.08397**
**3,4,5-Trimethoxycinnamic acid**	**10.68**	**239.09168**	**238.08441**
**3-Hexenedioic acid**	**3.59**	**145.04942**	**144.04214**
**3-Hydroxymethylglutaric acid**	**3.53**	**163.06006**	**162.05278**
**3-Indolepropionic acid**	**12.32**	**190.08703**	**189.07976**
**3-Methylindole**	**9.3**	**132.08086**	**131.07359**
**3-Methylxanthine**	**8.85**	**167.05661**	**166.04934**
**4-Aminohippuric acid**	**8.72**	**195.07649**	**194.06921**
**5-Hydroxy-L-tryptophan**	**9.54**	**221.09233**	**220.08505**
**5-Hydroxyindoleacetic acid**	**8.86**	**192.06586**	**191.05859**
**5-Hydroxylysine**	**13.29**	**163.1118**	**162.10453**
**9-Methyluric acid**	**8.85**	**183.05181**	**182.04454**
**Acetic acid**	**11.5**	**61.0283**	**60.02102**
**All-trans-retinoic acid**	**15.24**	**301.21685**	**300.20957**
**Allantoic acid**	**1.9**	**177.06127**	**176.054**
**Alpha-Tocopherol**	**20.31**	**431.38435**	**430.37708**
**Androstenedione**	**15.01**	**287.2006**	**286.19332**
**Androsterone**	**17.81**	**291.23018**	**290.2229**
**Benzamide**	**8.98**	**122.05785**	**121.05057**
**Benzoic acid**	**5.9**	**123.04392**	**122.03664**
**Biotin**	**9.31**	**245.09839**	**244.09111**
**Caffeine**	**9.79**	**195.08803**	**194.08075**
**Cinnamic acid**	**8.85**	**149.06018**	**148.0529**
**Creatine**	**2.39**	**132.07678**	**131.0695**
**Creatinine**	**2.37**	**114.06616**	**113.05888**
**D-Alanine**	**2.39**	**90.05527**	**89.04799**
**Deoxycholic acid glycine conjugate**	**14.1**	**450.32217**	**449.31425**
**DL-2-aminooctanoic acid**	**2.45**	**160.13322**	**159.12595**
**Elaidic acid**	**0.71**	**283.26283**	**282.25555**
**Estradiol**	**13.24**	**273.18528**	**272.178**
**Gamma-terpinene**	**16.95**	**137.13267**	**136.12539**

**Glycerophosphocholine**	**2.19**	**280.09216**	**257.10294**
**Glycine**	**3.47**	**76.03958**	**75.0323**
**Glycyl-L-leucine**	**9.25**	**189.12393**	**188.11666**
**Guanidine**	**2.66**	**60.05568**	**59.04841**
**Heptadecanoic acid**	**26.01**	**271.2634**	**270.25612**
**Hippuric acid**	**10.57**	**202.04784**	**179.05857**
**Homoveratric acid**	**10.93**	**197.07839**	**196.07112**
**Hyodeoxycholic acid**	**21.52**	**393.29695**	**392.28968**
**Indole**	**9.18**	**118.06528**	**117.05801**
**Indole-3-carbinol**	**9.44**	**148.07568**	**147.0684**
**Indoleacetic acid**	**11.62**	**176.0709**	**175.06363**
**Indolelactic acid**	**11.61**	**206.08113**	**205.07385**
**Isovalerylcarnitine**	**9.44**	**246.17081**	**245.16353**
**L-Acetylcarnitine**	**3.94**	**204.12305**	**203.11578**
**L-Arginine**	**2.67**	**175.11914**	**174.11186**
**L-Aspartyl-L-phenylalanine**	**9.91**	**281.1136**	**280.10633**
**L-Carnitine**	**2.63**	**162.11239**	**161.10511**
**L-Glutamic acid**	**3.52**	**148.06045**	**147.05317**
**L-Glutamine**	**10.02**	**147.07664**	**146.06937**
**L-Histidine**	**1.82**	**156.07692**	**155.06964**
**L-Kynurenine**	**9.4**	**209.09204**	**208.08477**
**L-Lysine**	**2.7**	**147.11326**	**146.10598**
**L-Methionine**	**3.84**	**150.05858**	**149.0513**
**L-Norleucine**	**4.76**	**132.10191**	**131.09463**
**L-Proline**	**2.67**	**116.0706**	**115.06333**
**L-Sorbose**	**9.67**	**181.07229**	**180.06501**
**L-Threonine**	**9.73**	**120.06559**	**119.05832**
**L-Tryptophan**	**9.82**	**205.09712**	**204.08984**
**L-Valine**	**2.68**	**118.08619**	**117.07892**
**Linoleic acid**	**17.79**	**281.24755**	**280.24027**
**m-Aminobenzoic acid**	**2.39**	**160.03472**	**137.04564**
**m-Coumaric acid**	**5.82**	**165.05461**	**164.04734**
**Medroxyprogesterone**	**16.47**	**345.24287**	**344.23559**
**N,N-Dimethylformamide**	**2.66**	**74.06024**	**73.05296**
**Nonadecanoic acid**	**29.91**	**299.29499**	**298.28772**
**Normetanephrine**	**2.33**	**184.09468**	**183.0874**
**Nutriacholic acid**	**21.43**	**413.26578**	**390.27682**
**o-Tyrosine**	**3.81**	**182.0814**	**181.07413**
**p-Octopamine**	**4.14**	**154.08392**	**153.07665**

**Paraxanthine**	**9.88**	**181.07204**	**180.06476**
**PC(16:0/16:0)**	**20.32**	**734.56788**	**733.5606**
**Phenylacetaldehyde**	**14.42**	**121.06472**	**120.05745**
**Phenylacetic acid**	**12.9**	**137.05962**	**136.05235**
**Pipecolic acid**	**1.79**	**130.08625**	**129.07898**
**Pregnenolone**	**19.15**	**317.24822**	**316.24094**
**Pregnenolone sulfate**	**15.32**	**397.20054**	**396.19326**
**Pyridoxamine**	**9.54**	**169.09724**	**168.08997**
**Pyroglutamic acid**	**3.76**	**130.04995**	**129.04267**
**Quinaldic acid**	**8.87**	**174.05556**	**173.04828**
**Retinal**	**17.58**	**285.22164**	**284.21436**
**Saccharopine**	**16.52**	**277.13545**	**276.12817**
**Sphinganine**	**13.15**	**302.30576**	**301.29849**
**Sphingosine**	**13.51**	**282.27948**	**299.28278**
**Stearic acid**	**24.6**	**285.27764**	**284.27037**
**Threonic acid**	**4.87**	**137.04577**	**136.03849**
**Thymol**	**17.45**	**151.11199**	**150.10471**
**Thyroxine**	**12.35**	**777.69442**	**776.68715**
**Traumatic acid**	**10.83**	**229.14156**	**228.13429**
**Trimethylamine**	**20.45**	**60.08098**	**59.0737**
**Urea**	**22.82**	**61.03982**	**60.03254**
**Uric acid**	**4.94**	**169.03565**	**168.02837**
**Uridine**	**14.87**	**245.07889**	**244.07161**
**Vitamin D3**	**22.01**	**385.34666**	**384.33938**

**Table S2:** Chemical structures of metabolites	
**Name**	**Chemical Structure**
**1,3-Dimethyluric acid**	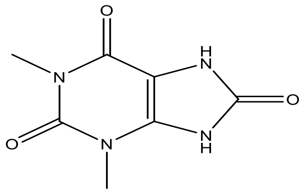
**1-Methylhistidine**	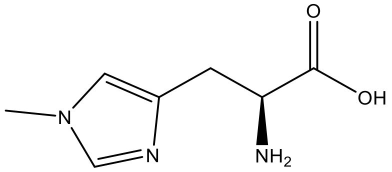
**17-Hydroxyprogesterone**	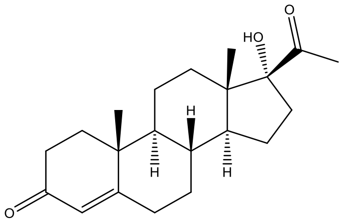
**2,4-Diaminobutyric acid**	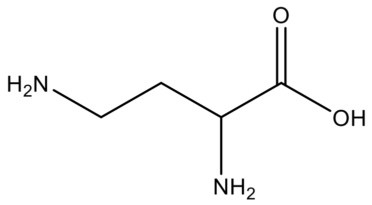
**2-Furoylglycine**	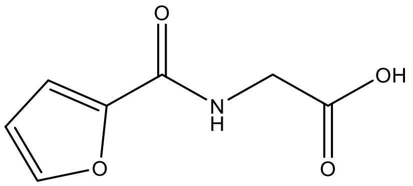
**2-Phenylbutyric acid**	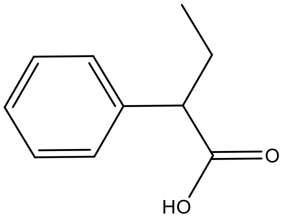
**3,4,5-Trimethoxycinnamic acid**	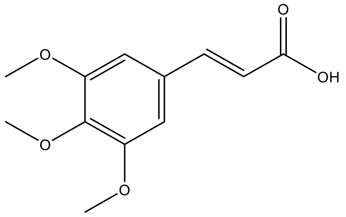

**3-Hexenedioic acid**	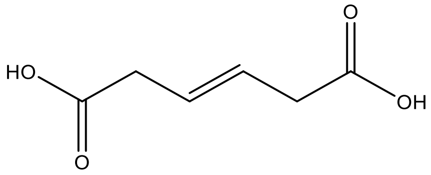
**3-Hydroxymethylglutaric acid**	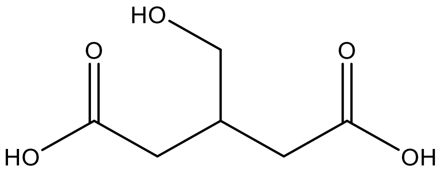
**3-Indolepropionic acid**	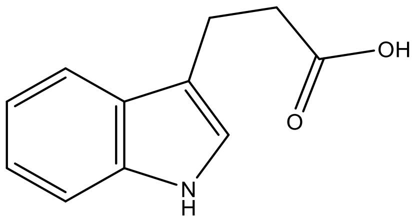
**3-Methylindole**	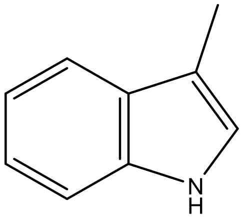
**3-Methylxanthine**	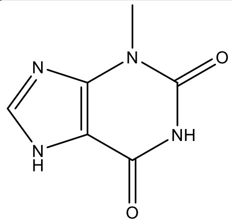
**4-Aminohippuric acid**	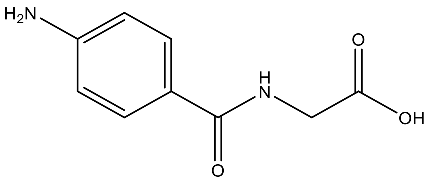
**5-Hydroxy-L-tryptophan**	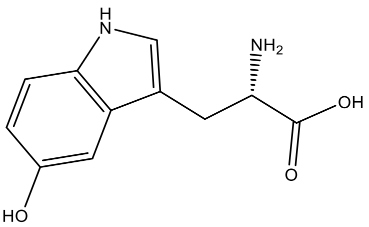

**5-Hydroxyindoleacetic acid**	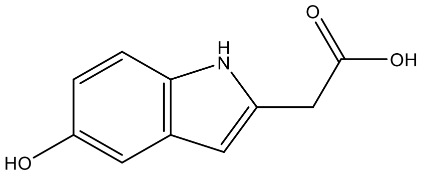
**5-Hydroxylysine**	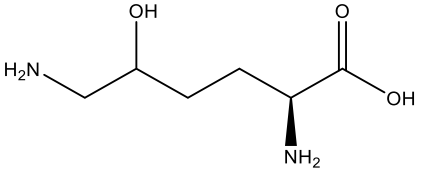
**9-Methyluric acid**	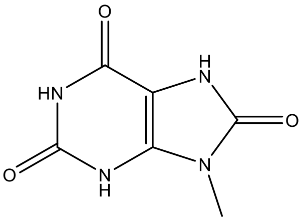
**Acetic acid**	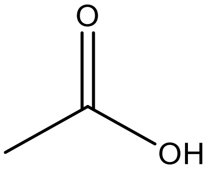
**All-trans-retinoic acid**	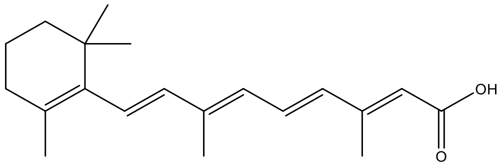
**Allantoic acid**	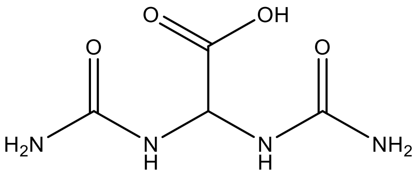
**Alpha-Tocopherol**	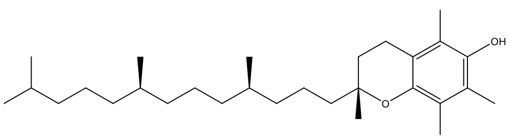

**Androstenedione**	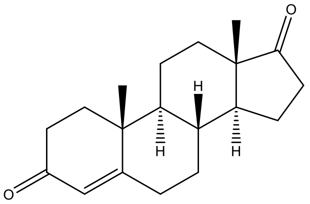
**Androsterone**	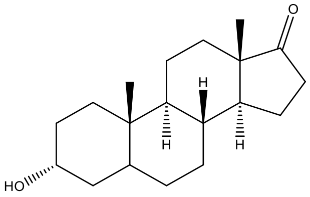
**Benzamide**	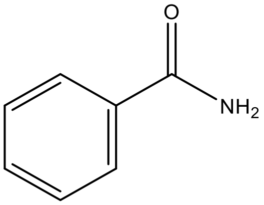
**Benzoic acid**	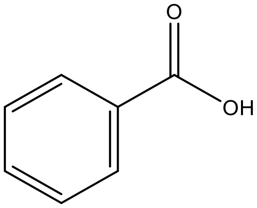
**Biotin**	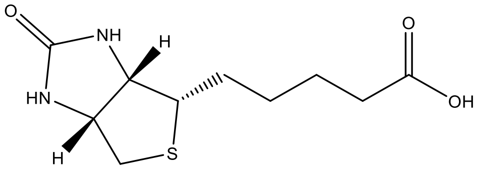
**Caffeine**	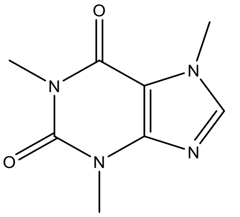
**Cinnamic acid**	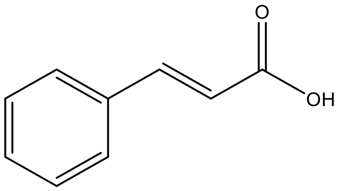

**Creatine**	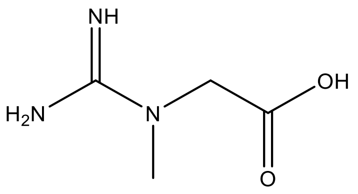
**Creatinine**	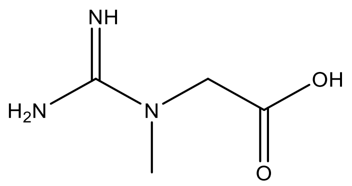
**D-Alanine**	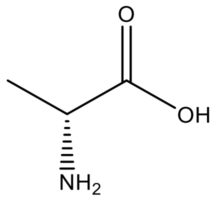
**Deoxycholic acid glycine conjugate**	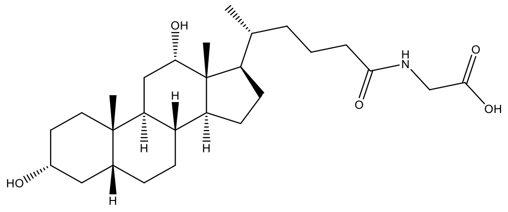
**DL-2-aminooctanoic acid**	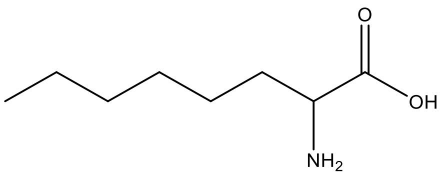
**Elaidic acid**	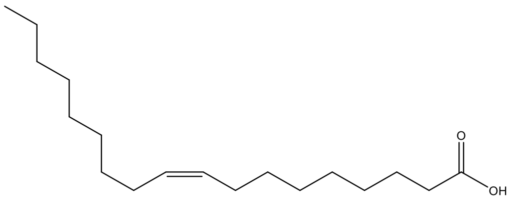
**Estradiol**	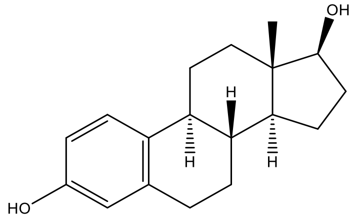

**Gamma-terpinene**	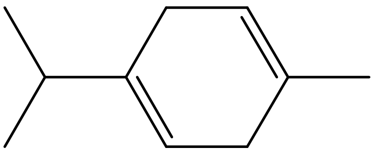
**Glycerophosphocholine**	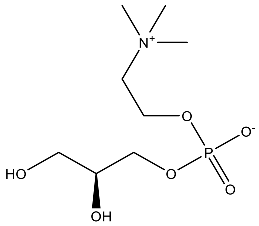
**Glycine**	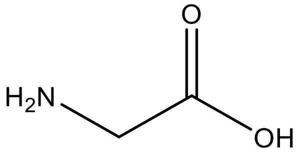
**Glycyl-L-leucine**	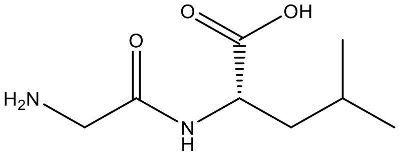
**Guanidine**	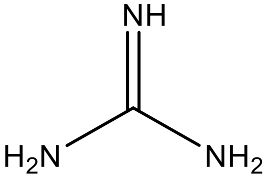
**Heptadecanoic acid**	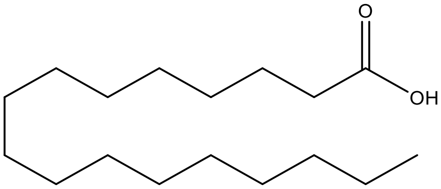
**Hippuric acid**	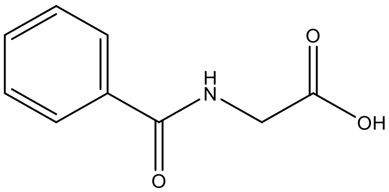

**Homoveratric acid**	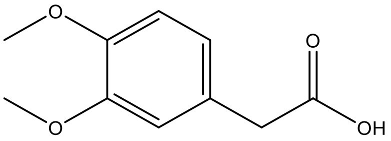
**Hyodeoxycholic acid**	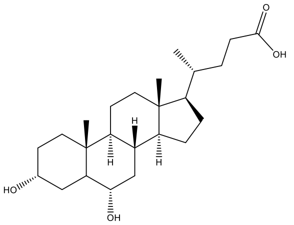
**Indole**	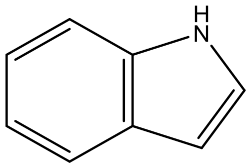
**Indole-3-carbinol**	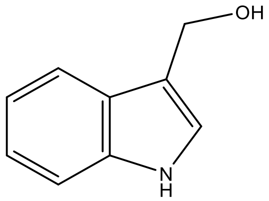
**Indoleacetic acid**	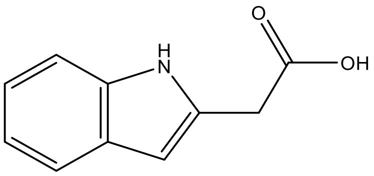
**Indolelactic acid**	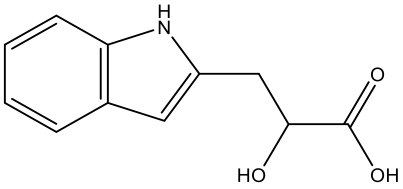
**Isovalerylcarnitine**	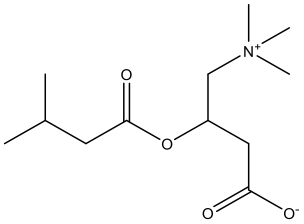

**L-Acetylcarnitine**	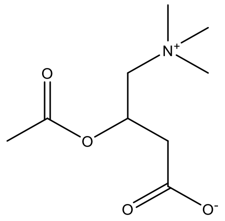
**L-Arginine**	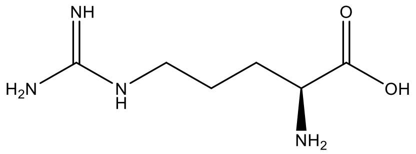
**L-Aspartyl-L-phenylalanine**	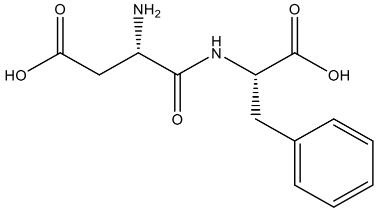
**L-Carnitine**	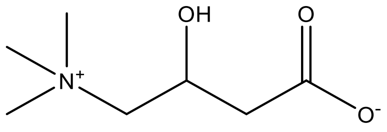
**L-Glutamic acid**	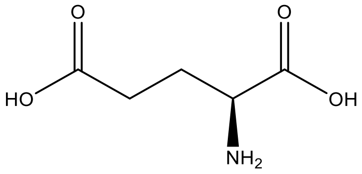
**L-Glutamine**	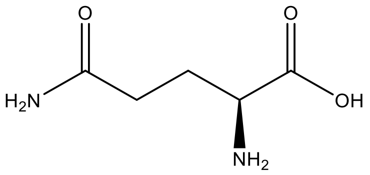
**L-Histidine**	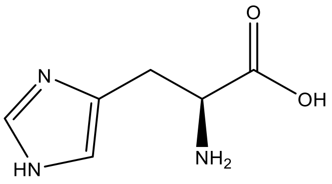

**L-Kynurenine**	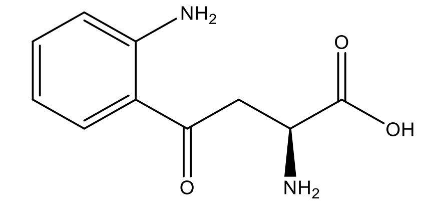
**L-Lysine**	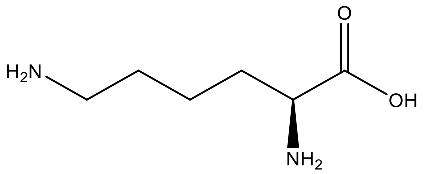
**L-Methionine**	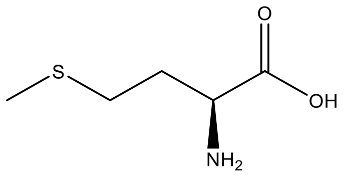
**L-Norleucine**	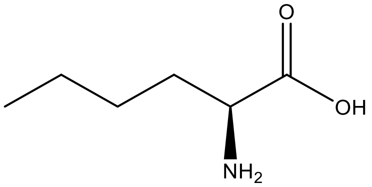
**L-Proline**	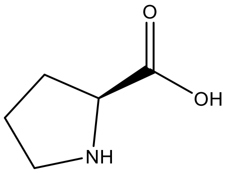
**L-Sorbose**	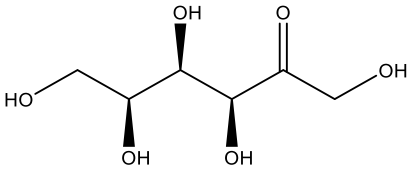
**L-Threonine**	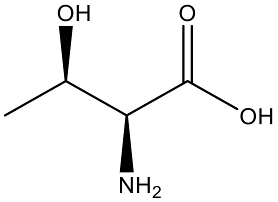

**L-Tryptophan**	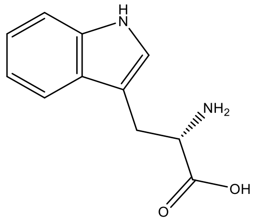
**L-Valine**	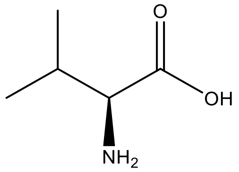
**Linoleic acid**	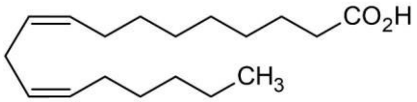
**m-Aminobenzoic acid**	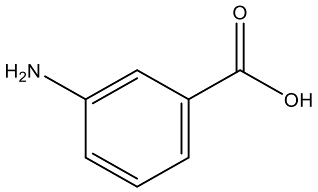
**m-Coumaric acid**	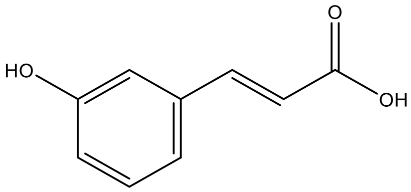
**Medroxyprogesterone**	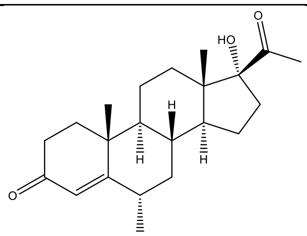
**N,N-Dimethylformamide**	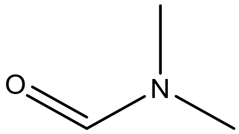

**Nonadecanoic acid**	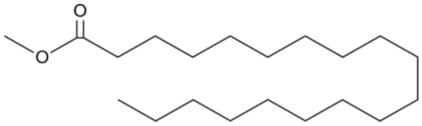
**Normetanephrine**	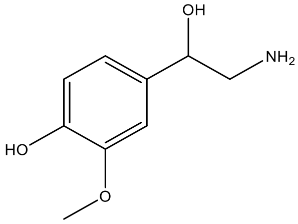
**Nutriacholic acid**	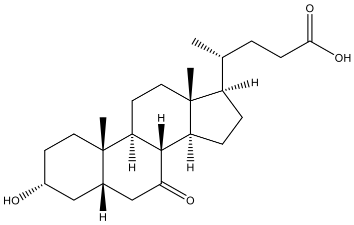
**o-Tyrosine**	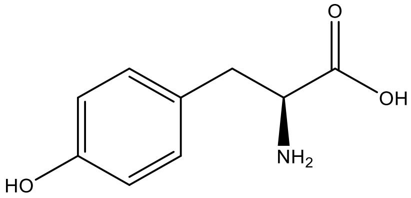
**p-Octopamine**	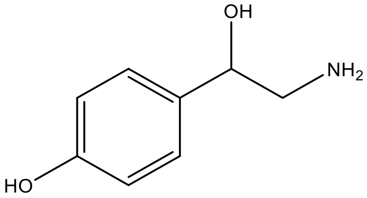
**Paraxanthine**	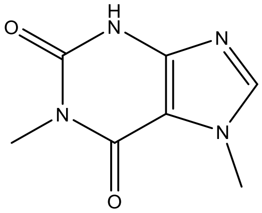
**PC(16:0/16:0)**	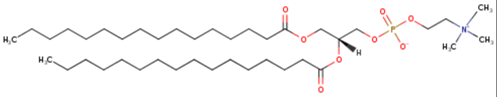

**Phenylacetaldehyde**	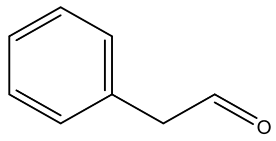
**Phenylacetic acid**	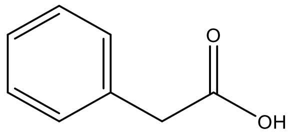
**Pipecolic acid**	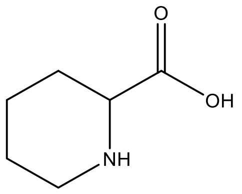
**Pregnenolone**	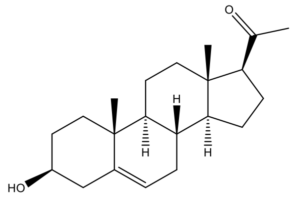
**Pregnenolone sulfate**	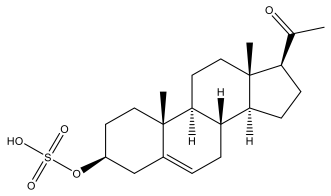
**Pyridoxamine**	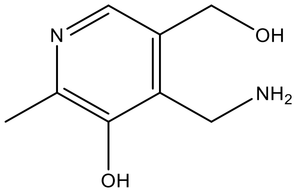
**Pyroglutamic acid**	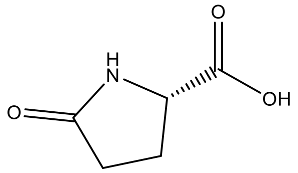

**Quinaldic acid**	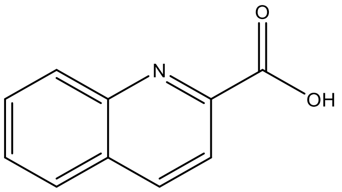
**Retinal**	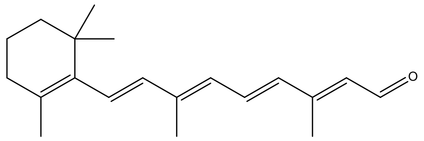
**Saccharopine**	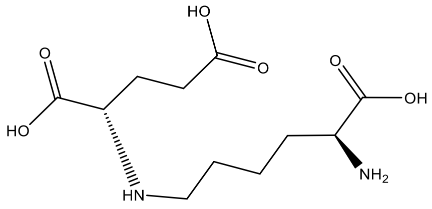
**Sphinganine**	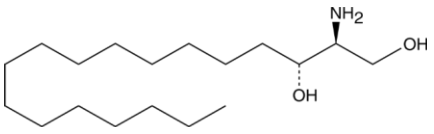
**Sphingosine**	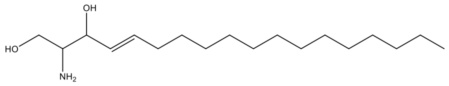
**Stearic acid**	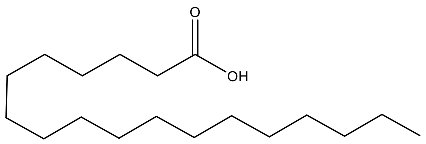
**Threonic acid**	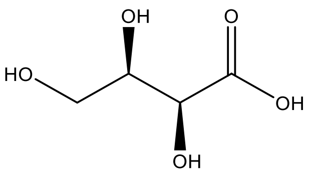

**Thymol**	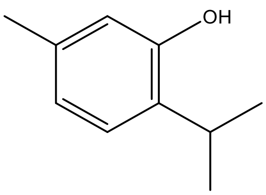
**Thyroxine**	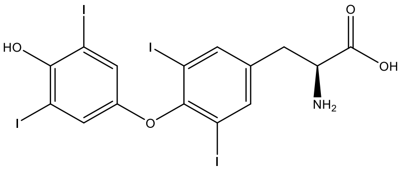
**Trimethylamine**	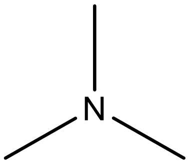
**Urea**	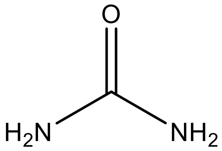
**Uric acid**	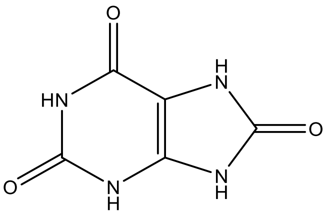
**Uridine**	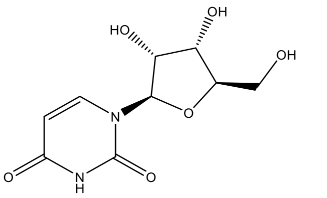
**Vitamin D3**	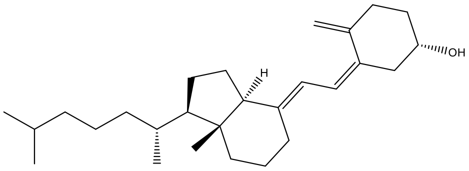

**Pantothenic acid**	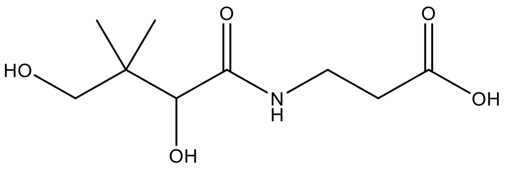
**Arginine**	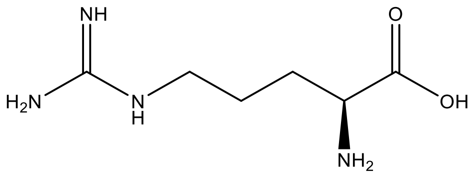
**Phenylalanine**	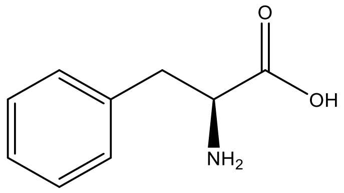
**Lysine**	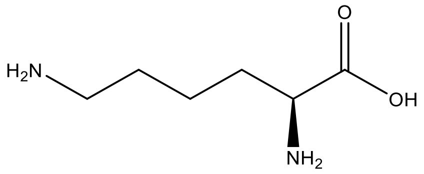
**Glutathione**	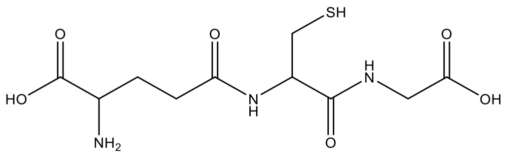
**Glyoxylate Dicarboxylate Conjugate**	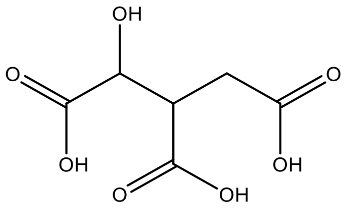
